# Perspectives on intestinal tapeworm infections: An evaluation of direct and indirect life-cycles with a special emphasis on species of *Hymenolepis*

**DOI:** 10.1016/j.crpvbd.2021.100023

**Published:** 2021-04-14

**Authors:** Akira Ito, Christine M. Budke

**Affiliations:** a Department of Parasitology, Asahikawa Medical University, Asahikawa 078-8510, Japan; b Department of Veterinary Integrative Biosciences, College of Veterinary Medicine & Biomedical Sciences, Texas A&M University, College Station, TX, USA

**Keywords:** *Hymenolepis nana*, *Hymenolepis microstoma*, Tailed cysticercoid, Tailless cysticercoid, Evolution from indirect to direct life-cycle

## Abstract

Numerous experimental studies have been conducted on the rodent tapeworm, *Hymenolepis microstoma*. In contrast, less is known about the life-cycle and immunobiology of the zoonotic dwarf tapeworm, *Hymenolepis nana*. However, *H. nana* appears to be unique in that; (i) it can complete its entire life-cycle within a single mammalian host, and (ii) cysticercoids that develop in beetle intermediate hosts are tailed, while those that develop in the intestinal tissue of mammals are tailless. This is in contrast to all other *Hymenolepis* spp., which only appear to develop tailed cysticercoids in beetles or experimentally infected immunodeficient rodents. Even though *H. microstoma* and *H. nana* are phylogenetically much closer to each other than to *Hymenolepis diminuta*, when mice with severe combined immunodeficiency (SCID) were inoculated with *H. microstoma* eggs, hatched oncospheres invaded the intestinal tissue and developed into infective tailed cysticercoids in approximately 11 days. Therefore, *H. nana* appears to be truly unique in its ability to develop tailed cysticercoids in beetles and tailless cysticercoids in mammals. These unique evolutionary characteristics are discussed in relation to other cyclophyllidean cestodes, including *Taenia solium* and *Echinococcus* spp.

## Highlights

• *Hymenolepis nana* is unique in that it can complete its entire life-cycle within a single mammalian host.
• The ability to develop tailless cysticercoids in mammals may be an evolutionary adaption for *H. nana*.
• Adult cestode lifespan may be a good indicator as to whether or not a particular species can have a direct life-cycle.

## Graphical abstract

**Figure:**
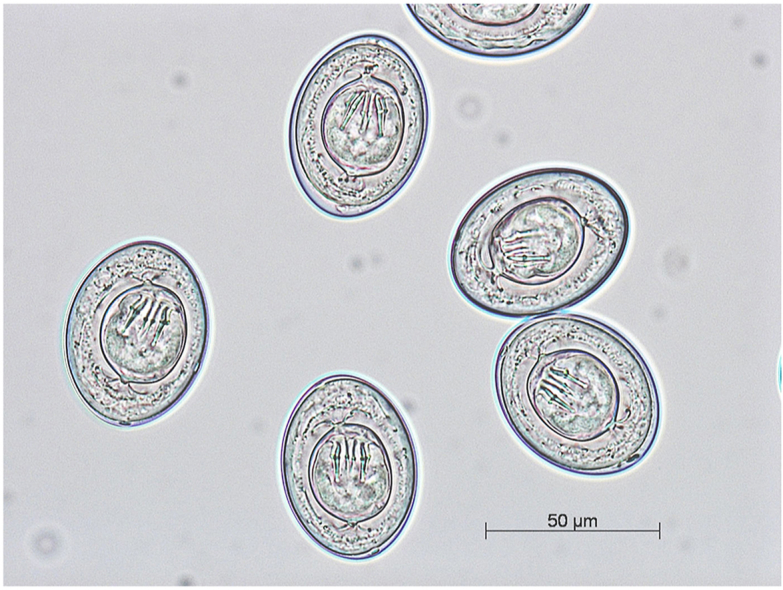

## 1 Introduction

Cestodes belonging to the genus *Hymenolepis* use arthropods, most commonly beetles belonging to the species *Tribolium* or *Tenebrio*, as intermediate hosts. These beetles harbor cysticercoid larvae. Rodents and other small mammals act as definitive hosts, which harbor adult tapeworms in the intestine or bile duct, as is the case for *Hymenolepis microstoma* (the mouse bile duct tapeworm). *Hymenolepis nana* (the mouse tapeworm) and *Hymenolepis diminuta* (the rat tapeworm) are well known zoonotic tapeworms (Arai, 1980). Among the three species, *H. diminuta* is phylogenetically distant from *H. nana* and *H. microstoma* (Okamoto et al., 1997; Jarošová et al., 2020). Adult worms of most species in this genus live for one to two years, which typically corresponds to the lifespan of the host rodent (Arai, 1980; Andreassen, 2010). The exceptions include *H. nana*, which survives less than one month and possibly *H. microstoma*, which survives in the bile duct for at least six months but likely less than a year. Therefore, the *H. nana* lifespan appears to be shortest followed by *H. microstoma* (Ito & Smyth, 1987; Cunningham & Olson, 2010).

The difference in lifespan is possibly related to *H. nana* evolutionarily establishing a direct life-cycle (i.e. *via* ingestion of parasite eggs) *versus* only the indirect life-cycle (i.e. *via* ingestion of cysticercoids) found in all other species of *Hymenolepis*, including *H. microstoma*. Due to this difference, *H. microstoma* does not appear to be a good model to study *H. nana* and other taeniids that have alternative direct life-cycles consisting of intestinal tapeworm infection followed by larval stage development in parenteral tissue after egg ingestion. In many ways, *H. nana* has more similarities with the zoonotic taeniids, *Taenia solium* and *Echinococcus* spp. than with other members of the genus *Hymenolepis*. For example, while *T. solium* completes its entire life-cycle between pigs (intermediate hosts) and humans (definitive host), humans can also be infected with eggs through autoinfection or from other tapeworm carriers. Among the three human-infecting *Taenia*, which include *T. solium*, *Taenia saginata*, and the ‘Asian *Taenia*’ (*T. saginata asiatica*) (Fan et al., 1995; Okamoto et al., 2010; Yamane et al., 2012; Ito & Budke, 2021), *T. solium*, exclusively causes cysticercosis in humans. Although less common, *Echinococcus* infection in the definitive host may result in infection with the larval stage of the parasite, causing cystic or alveolar echinococcosis (Ishino, 1941; Weiss & Köhler, 2010; Konyaev et al., 2012; Gottstein et al., 2014; Conraths & Deplazes, 2015; Frey et al., 2017; Antolová et al., 2018; Zajac et al., 2020). This paper focuses on the establishment of indirect and direct life-cycles for cyclophyllidean cestodes and builds upon previous reviews of these parasites (Ito, 2015, 2016; Ito & Budke, 2021).

## 2 *Hymenolepis nana*

Definitive hosts for the *H. nana* tapeworm include rodents and other small mammals and humans. These hosts are infected through ingestion of eggs (direct cycle) or metacestodes (cysticercoids) located in beetles and other intermediate hosts (indirect cycle). All developmental stages, including oncospheres, cysticercoids, and adult tapeworms appear to be immunogenic, resulting in stage-specific immunity to reinfection (Ito, 1984; Ito & Onitake, 1986; Ito & Smyth, 1987). When a definitive host becomes infected *via* the direct cycle, oncospheres invade the intestinal tissue and the host becomes immune to reinfection within a few days (Hunninen, 1935; Heyneman, 1962; Weinmann, 1966; Okamoto, 1970; Okamoto & Koizumi, 1972; Asano et al., 1986; Asano & Okamoto, 1992; Asano & Muramatsu, 1997). A host may become immune after only a single oncosphere invades the intestinal wall ( Ito & Yamamoto, 1976). When a definitive host is infected with *H. nana* eggs, the resulting mature tapeworms produce eggs. However, there is no autoinfection due to acquired immunity to the parasite eggs, which occurs within 4 days after primary infection with eggs. Oncospheres from reinfection may invade the intestinal tissue, but do not develop into cysticercoids ( Ito & Smyth, 1987).

Similar to the direct infection cycle, definitive hosts infected *via* ingestion of *H. nana* cysticercoids, found in beetle intermediate hosts, produce adult worms that release eggs. However, when a definitive host is experimentally infected with cysticercoids, either from beetles or other rodents, autoinfection resulting in large second-generation stunted worms can occur in some strains of mice (Ito & Kamiyama, 1984; Ito, 1985; Ito et al., 1988) (see figure 1 in Ito, 2015). Adult tapeworm longevity and autoinfection are affected by the host immune response to the lumen phase of *H. nana* cysticercoids and adult tapeworms, but the timing of this response is highly variable among mouse strains. Usually, within one month of infection, the adult tapeworms become stunted and disappear ( Ito & Smyth, 1987). However, adult worm longevity is variable among hosts. The mechanism by which this stunting occurs remains unknown.

There are several *H. nana* strains that do not appear to cause autoinfection (Ito, 1982; Ito et al., 1988). This may be explained by the rapid onset of immunity to the parasite’s lumen phase before eggs are released from cysticercoid-derived adult tapeworms. However, other unknown factors may be involved. The indirect cycle can be established in small mammal definitive hosts by feeding them cysticercoids. Some strains of *H. nana* do not mature in rats, including congenitally athymic nude rats (Ito, 1983; Ito & Kamiyama, 1984; Ito, 1985), but instead the rats develop cysticercoids. There does not appear to be a difference in antigenicity of *H. nana* cysticercoids developed in beetles compared to those developed in small mammals (Ito & Onitake, 1987). An interesting phenomenon is that *H. nana* cysticercoids that develop in mammals (e.g. mice, rats, hamsters) have no tails and have been named ‘tailless cysticercoids’, while those in beetles have tails and are called ‘tailed cysticercoids’. The tailless variety of cysticercoids does not appear to be present in any other hymenolepidid species.

Infective *H. nana* cysticercoids develop in mouse intestinal tissue within 4 days and escape into the intestinal lumen to develop into adult tapeworms. However, in immunodeficient mice, some oncospheres migrate to the liver similar to certain taeniid species and some cysticercoids become established in deeper intestinal tissues and the liver where they remain as balloon-like cysticercoids (Ito & Kamiyama, 1984; Ito, 1985; Ito, 2015) (see figure 1 in Ito, 2016). The presence of cysticercoids in the mouse liver suggests a possible alternative cycle by cannibalism if the parasite can complete its life-cycle outside of the mouse intestine.

All species belonging to the genus *Hymenolepis* mature into adult tapeworms in small mammals and primarily use beetles as intermediate hosts. *Hymenolepis nana* is more common in mice than in rats (Ogura, 1936; Soh et al., 1961). For example, an isolate first maintained in mice located in a laboratory in Tokyo, Japan, more than 50 years ago does not mature in rats (Okamoto, 1970; Ito & Kamiyama, 1984; Ito, 1985). Molecular studies will be informative to further evaluate any differences in mouse-adapted *versus* rat-adapted *H. nana* isolates. All *H. nana* isolates are also known to be infective to humans (Weinmann, 1966). A recent study conducted in Mexico where both *H. nana* and *H. microstoma* are known to occur revealed that all hymenolepidid eggs identified in children were *H. nana* (see Panti-Maya et al., 2020). Thus far, *H. nana* and *H. diminuta* are the only known truly zoonotic species in the genus *Hymenolepis*. However, other species may occasionally infect humans as evidenced by the recovery of an *Hymenolepis hibernia* adult worm from a 52-year-old Tibetan woman (Nkouawa et al., 2015).

## 3 *Hymenolepis microstoma*

In contrast to *H. nana*, more is known about *H. microstoma* and the parasite’s full genome has been analyzed (Cunningham & Olson, 2010; Tsai et al., 2013). *Hymenolepis microstoma* adults, whose scolices attach to the bile duct, can survive approximately six months without reinfection (Jarošová et al., 2020). In this species, fully mature tailed cysticercoids develop approximately 11 days after egg inoculation in mice with severe combined immunodeficiency (SCID) (Andreassen et al., 2004). When mice infected with *H. microstoma* were housed in the same cage with uninfected mice, the uninfected mice eventually showed antibody responses to *H. microstoma* oncospheres (Ito et al., 1989). This suggests that even though *H. microstoma* has not been shown to have a direct life-cycle, oncospheres may still hatch and invade intestinal tissue (Onitake et al., 1990).

Gene expression, in *H. microstoma*, allows it to hatch in the intestines of mammalian hosts, developing into tailed cysticercoids in immunodeficient mice (Andreassen et al., 2004). Experimental infection of congenitally athymic nude mice with *H. microstoma* eggs resulted in few cysticercoids in the intestinal tissue. When these SCID mice were then infected with cysticercoids, they produced first generation tapeworms in addition to a large number of tailed cysticercoids derived from autoinfection (second generation). In comparison, when congenitally athymic NMRI-*nu* mice infected with cysticercoids, which later became adult *H. microstoma*, were allowed to eat their own feces containing tapeworm eggs, the oncospheres penetrated the intestinal tissue and developed into cysticercoids. After excysting, the parasites grew into adult worms in the lumen of the small intestine and bile duct. The same outcome occurred when NMRI-*nu* mice, non-obese diabetic SCID (NOD/Shi-*scid*) mice, and NOD/Shi-*scid*, IL-2 Ry^null^ (NOG) mice were orally inoculated with *H. microstoma* eggs (Andreassen et al., 2004).

In summary, differences between *H. microstoma* and *H. nana* cysticercoid development in mouse intestinal tissues include: (i) the time course for development of infective cysticercoids was approximately 11 days for *H. microstoma*, but only 4 days for *H. nana*; and (ii) *H. microstoma* cysticercoids that developed in mice had tails, while those of *H. nana* had no tails. Therefore, *H. nana* appears to be unique among *Hymenolepis* tapeworms in its ability to adapt to direct infection, with resulting unique morphologic characteristics (i.e. the production of tailless cysticercoids).

## 4 Comparison of the Hymenolepididae and Taeniidae

Experimental studies in rodents have shown that host immunity can occur after a single egg has invaded the intestinal wall for both *H. nana* (see Ito & Yamamoto, 1976) and *Taenia taeniaeformis* (see Ito & Hashimoto, 1993). In addition, the speed at which differentiation from oncosphere to metacestode occurs may contribute to the variability in immune response between taeniid species (Okamoto et al., 2005). Unlike taeniid eggs that require mammalian bile salts to hatch, hymenolepidid oncospheres are easily hatched and activated *in vivo* and *in vitro* without the use of a stimulative solution (Laws, 1968). Physiological or mechanical stimulation is sufficient to activate and hatch the oncospheres. This difference may be related to the short lifespan of *Hymenolepis* spp. compared to some taeniids (Yoshino, 1934; Garcia et al., 2003; Lightowlers, 2010; Li et al., 2019; Zein et al., 2019; Ito et al., 2020; Ito & Budke, 2021).

Among the taeniids, *T. solium* is exceptional in that human infection regularly occurs through ingestion of parasite eggs (resulting in cysticercosis) or cysticerci (resulting in taeniasis). Infection with *Echinococcus* spp. eggs has been reported in carnivorous and omnivorous definitive hosts, resulting in echinococcosis (Ishino, 1941; Konyaev et al., 2012; Corsini et al., 2015; Ito, 2016; Fley et al., 2017; Antolová et al., 2018). However, at this time, there is no indication that other human-infecting *Taenia* spp. (*T. saginata* and ‘Asian *Taenia*’) can result in human cysticercosis. While there has been some conjecture regarding human cysticercosis caused by ‘Asian *Taenia*’, no evidence is currently available to support this hypothesis (McManus, 1996, 2006; Ito & Budke, 2021). Based on molecular findings, some researchers believe ‘Asian *Taenia*’ to be an intraspecies variant of *T. saginata* and *T. saginata asiatica* (Zarlenga et al., 1991; Bowles & McManus, 1994; McManus & Bowles, 1994; Fan et al., 1995; Gasser et al., 1999; Okamoto et al., 2007, 2010; Yamane et al., 2012, 2013), while others believe it to be a unique species, *Taenia asiatica* (see Eom & Rim, 1993). Regardless, serological screening in populations located in Sichuan, China, where *T. solium*, *T. saginata*, and ‘Asian *Taenia*’ are sympatrically distributed, appears to further support the idea that cysticercosis is exclusively caused by *T. solium* (see Li et al., 2019).

Overall, adult parasite lifespan may be a good indicator as to whether or not the species may have a direct life-cycle. For example, *H. nana* is known to have a short life-span, while there is some evidence that the *T. solium* lifespan is also short, with a hypothesized lifespan less than five years (Yoshino, 1934; Garcia et al., 2003; Lightowlers, 2010;Li et al., 2019; Zein et al., 2019; Ito et al., 2020; Ito & Budke, 2021). In contrast, the other human taeniids, *T. saginata* and ‘Asian *Taenia*’, are known to live as long as 50 years (Li et al., 2019; Zein et al., 2019).

## 5 Perspectives

The question arises of what are the next steps in trying to better understand infection by *Hymenolepis* spp. and other cyclophyllidean cestodes. First, it would be beneficial to experimentally infect mice or rats with *H. diminuta* eggs and see if pathogen-free rodents kept in the same cage develop an antibody response (Ito et al., 1989), similar to what was seen with *H. microstoma*. If uninfected rodents show antibody responses to oncosphere antigens, the next step would be to duplicate the experiment using congenitally athymic nude rats or SCID rats. This type of study can also be applied to *Taenia* spp. and *Echinococcus* spp. (Nakaya et al., 2006; Nakao et al., 2007, 2013a, b). Secondly, if other cyclophyllidean cestode species besides *H. nana*, *T. solium*, and *Echinococcus* spp. are found to have relatively short lifespans in the definitive hosts, studies will need to be conducted to evaluate if definitive hosts can also become infected *via* the ingestion of parasite eggs. These types of studies may help clarify why the larval stage of *T. solium* is able to develop in the definitive host (Hoberg et al., 2001; Nakao et al., 2002), while this is not possible for *T. saginata* and ‘Asian *Taenia*’ (Ito et al., 2003; Li et al., 2019; Ito & Budke, 2021). A final research theme to evaluate in greater detail is the mechanism for the rapid onset (within a few days) of reinfection with eggs from *H. nana* (see Okamoto, 1970; Okamoto & Koizumi, 1972; Asano et al., 1986; Asano & Okamoto, 1992; Asano & Muramatsu, 1997) and other species in the family Taeniidae. This rapid onset appears to be independent from antibody responses and requires additional research beyond what has been conducted for vaccine development (Rickard & Williams, 1982; Johnson et al., 1989; Ito et al., 1991; Lightowlers et al., 1993).

## Funding

There was no funding associated with this work.

## CRediT author statement

AI prepared the initial draft. CMB and AI edited the manuscript for content and language.

## Declaration of competing interests

The authors declare that they have no competing interests.
